# “How I whiten my teeth”: YouTube™ as a patient information resource for teeth whitening

**DOI:** 10.1186/s12903-020-01172-w

**Published:** 2020-07-01

**Authors:** Huseyin Simsek, Suleyman Kutalmış Buyuk, Ebru Cetinkaya, Mubin Tural, Murside Seda Koseoglu

**Affiliations:** 1 Department of Pediatric Dentistry, Faculty of Dentistry, Ordu University, Ordu, Turkey; 2 Department of Orthodontics, Faculty of Dentistry, Ordu University, Ordu, Turkey

**Keywords:** Dental bleaching, Internet, Social media, Video analysis, YouTube

## Abstract

**Background:**

YouTube™ is the world’s second most popular website after Google on the Internet. The aim of this study was to assess the quality and content of information YouTube™ videos for patients seeking information about teeth whitening.

**Methods:**

The keyword ‘teeth whitening’ was searched on YouTube™. YouTube™ was filtered by the relevance, and the first 100 videos that met the inclusion criteria were evaluated. The included videos were analyzed for views, duration, time since video upload, likes/dislikes, number of comments, source, material types (dental, natural, and other). Also, video purpose was analyzed under nine categories (definition, material preparation, the procedure of application, material comparison, before/after comparison, symptoms, post-op experience, commercial, educational). Each video was classified according to the quality of information content as ‘good’, ‘moderate’, or ‘poor’. The Kruskal-Wallis test, Fischer’s Exact test and Spearman correlation analyses were performed.

**Results:**

Most videos were uploaded by laypersons (60.0%). The definition of teeth whitening was the most commonly covered topic (74.0%), followed by the procedure of application (54.0%), and post-op experience (36.0%). Only 12% of videos were classified as having good information quality content, 53% moderate, and 35% were rated as poor information content. Poor-information content videos had a significantly higher number of viewing rates than the other groups (*P* = 0.002), besides the duration was significantly higher in poor-information content videos (*P* =0.002). There was a significant relationship between the quality of video information and material types (*P* <0.001).

**Conclusions:**

YouTube™ should not be used as a thoroughly reliable and accurate source for patient information about teeth whitening. More informative and reliable content YouTube™ videos about teeth whitening should be uploaded by professionals.

## Background

The Internet offers an attractive and useful platform for patients seeking information about their health [1]. In addition to face-to-face and patient-clinician interaction, nowadays Internet is one of the most important sources of information in dentistry. Due to the patients’ hesitation to ask the doctor face to face questions, long waiting times of the patients in the clinics and today’s COVID-19 virus infectiousness, it has become easier to get medical information on the Internet and encourages people to seek information in social media platforms [2]. More than 80% of Internet searching activities are for medical information and support [3]. The Internet is also used by professionals and laypersons to share experiences and knowledge [1, 4]. The Internet is a valuable resource for patients to get health information [5]. While health care professionals continue to be the most important source of information in directing the decisions of a patient, the impact of the information on the Internet is clearly visible [6].

YouTube™ is one of the most visited websites by patients who want to access medical information, and the YouTube™ website is the world’s second most popular website after Google on the Internet. The YouTube™ was established in 2005 as a video sharing website. Almost 5 billion videos are watched on YouTube™ a day, and an average user spends an average of 13 min 50 s on YouTube™ a day, nowadays [7, 8]. The YouTube™ videos are not reviewed by a reviewer by the nature of this platform, and the videos can be uploaded from a variety of sources and possibly of variable quality [9]. However, YouTube™ videos are entirely based on the principle of freedom of expression, and mostly unregulated [10]. This means that video sources may potentially contain inaccurate information. Most studies agree that YouTube™ includes scientifically incorrect and sometimes misleading health-related details that could harm patients’ health [11, 12].

Patients are not only willing to have a pleasant smile, but also to have more white teeth. They are not only aware of the stained teeth because they are also dissatisfied with the color of the teeth [13, 14]. Alkhatib et al. [15] reported that most of the respondents were dissatisfied with their tooth color in the United Kingdom. In a pediatric study conducted in the United States, 20% of parents and 31% of children reported that they were dissatisfied with tooth color [16]. Several studies showed that tooth color dissatisfaction is commonly reported in many different countries ranging from 32.3% to 64.1% [17–19]. Tooth whitening is one of the most popular cosmetic dental procedures for patients, and this procedure can be done with different materials in the dental office or by the patient at home [13].

As medical and dental professionals recognize the impact of YouTube™ as a patient information source, a few studies have been published analyzing the nature and quality of information available on YouTube™ [3, 12, 20]. However, no studies have investigated YouTube™ content about teeth whitening. Therefore, the aim of present study was to evaluate the content and quality of most relevant YouTube™ videos about teeth whitening.

## Methods

**Fig. 1:**
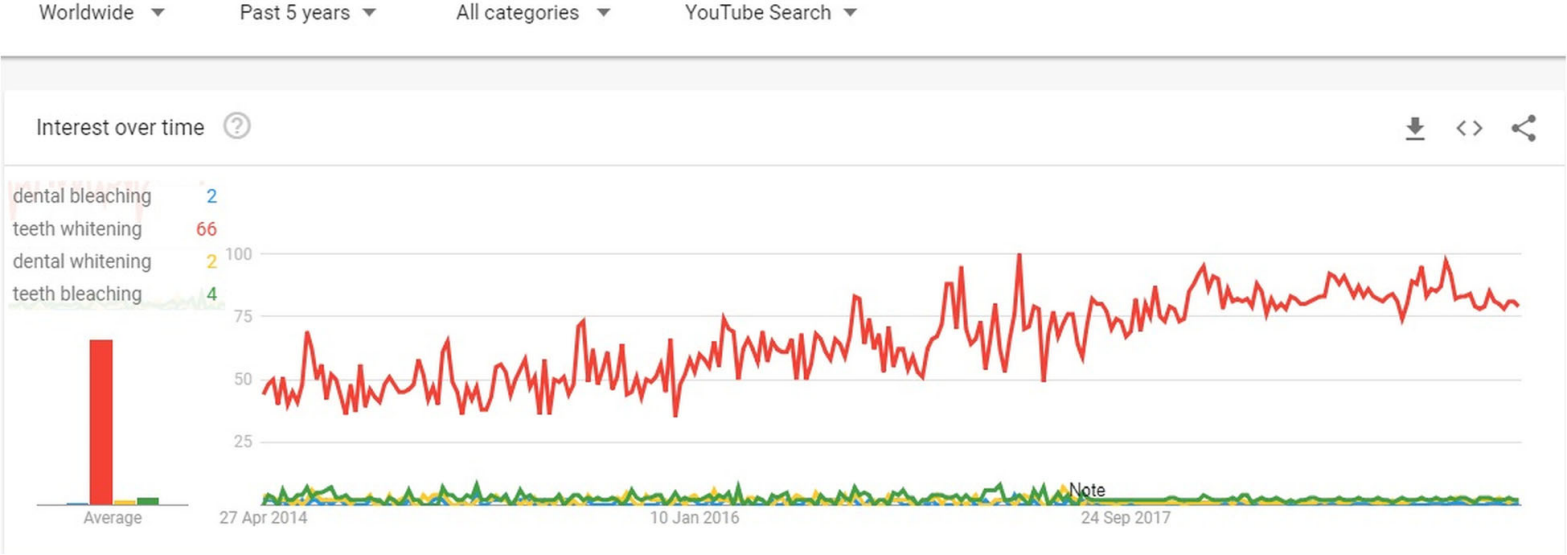
Interest rates in different phrase over time in Google Trends

YouTube™ was searched using the keyword ‘teeth whitening’ on 17 March 2019 by one of a researcher of present study (E.C.). The most commonly used terms were determined as ‘teeth whitening’, ‘dental bleaching’, ‘tooth whitening’ and ‘dental whitening’ in this topic. The search parameters have been limited to the last five years and ‘Worldwide’ settings, and the term ‘teeth whitening’ was used to search YouTube™, which was the most commonly used search term for ‘teeth whitening’ on the Google Trends application (Fig. 1). No ethical committee approval is required, since this study is performed on the publicly available Internet data.

The only search filter used was the ‘sort by relevance’, which is the default filter for a typical YouTube™ search. The new user account was created, computer history and cookies were deleted. The results of the search query are listed according to the relevance of the videos; this is determined by the YouTube™ website by combining the factors that include how many views, ratings, and upload dates. Non-English videos, songs, and conferences were excluded from the study. According to these criteria, the search results were limited to the first 100 YouTube™ videos in this study. The findings of previous studies have shown that most of YouTube™ users scan the first three pages several times a day, with more than 90% not interested in other pages [21].

Each video was evaluated for the following features: (1) number of views, (2) duration in minutes, (3) the total number of ‘likes’ and ‘dislikes’, (4) days since upload, (5) number of comments. Viewers’ interaction was calculated using the formulas, interaction index; [(number of likes-number of dislikes/total number of views) * 100%] and viewing rate; [(number of views/number of days since upload) * 100%]. The content of the videos was categorized into nine different groups: (1) definition, (2) description of material preparation, (3) procedure of application, (4) material comparison, (5) before/after comparison, (6) symptoms, (7) post-op experience, (8) commercial, (9) educational.

Videos were categorized into four groups according to their sources: (1) dentist/specialist, (2) clinic/hospital/university, (3) layperson, or (4) other. All videos were also categorized three basic groups according to the used material type: (1) dental materials (hydrogen peroxide, carbamide peroxide, toothpaste, mouthwash), (2) natural product (milk, lemon, tomato, apple cider vinegar, strawberry, turmeric, coconut oil, clove, carrot), (3) Other (activated carbon, petroleum jelly, carbonate). The information quality was categorized as good, moderate, poor based upon Hegarty et al. [22]. YouTube™ videos with no incorrect information, noteworthy quality and flow, most of the relevant information is included; excellent usefulness for patients were evaluated as good-information content. The videos moderate quality, sub-optimal flow; some critical information is adequately discussed, however, others poorly presented, somewhat useful for patients were evaluated as moderate-information content, and the videos with poor quality, reduced flow of the video, misleading information, no usefulness for patients were evaluated as poor-information content.

Statistical analysis was performed with statistical software (version 20, SPSS Inc., Chicago, IL, USA). Kruskal–Wallis Test was used to determine the differences between good, moderate, and poor information content videos and Spearman correlation coefficient was used to examine possible correlations of viewing rate and viewers’ interaction index with information quality. Fischer’s Exact test was used to evaluate differences between information quality and material groups. The information quality of all videos was reviewed by a second researcher (M. T.) to assess inter-rater reliability. Twenty videos were randomly selected and reviewed by the same authors after 1 month first evaluation. Kappa correlation coefficients were calculated to determine intra-rater and inter-rater reliability. The significance level was set at 5%.

## Results

The descriptive statistics of the evaluated 100 YouTube™ video demographics are presented in Table 1. The mean length of YouTube™ videos on teeth whitening was 5.83 min. The mean total number of views was 2,189,818.57. The mean viewing rate was 852,976.06. The overall mean number of ‘likes’ was 16,126.40, whereas the whole mean number of ‘dislikes’ was 1404.77. The mean number of comments was 786.29 (Table 1).

**Table 1:** Descriptive data of the YouTube™ videos about the teeth whitening

Video Features	Mean	Standard Deviation	Minimum	Maximum
Number of Views	2,189,818.57	6,705,555.36	69.00	42,140,927.00
Number of Likes	16,126.40	40,526.085	0.00	301,854.00
Number of Dislikes	1404.77	4372.50	0.00	27,221.00
Duration (minute)	5.83	3.27	0.42	15.49
Viewing Rate	852,976.06	4,517,448.14	805.49	44,413,715.07
Interaction Index	1.33	1.53	0.00	8.70
Number of Comments	786.29	2150.70	0.00	19,778.00

Most YouTube™ videos on teeth whitening were uploaded by laypersons (60%, n = 60). The definition of teeth whitening was the most commonly covered topic (74.0%), followed by a procedure of application (54.0%), post-op experience (36.0%), before/after comparison (29.0%), and process of material making (29.0%) (Table 2).

**Table 2:** Distribution of YouTube™ video characteristics in different information content video groups

Video Characteristics	Poor-Information Content Videos (***n*** = 35)	Moderate-Information Content Videos (***n*** = 53)	Good-Information Content Videos (***n*** = 12)	Total
*Video Source*	*n* (%) *n* (%) *n* (%) *n* (%)			
Clinic/Hospital/University	0 (0.0)	2 (3.8)	3 (25.0)	5 (5.0)
Dentist/Specialist	0 (0.0)	2 (3.8)	7 (58.3)	9 (9.0)
Layperson	17 (48.5)	41 (77.4)	2 (16.7)	60 (60.0)
Other	18 (51.4)	8 (15.1)	0 (0.0)	26 (26.0)
*Video Purpose*				
Definition	18 (51.4)	47 (88.7)	9 (75.0)	74 (74.0)
Procedure (Material making)	22 (62.9)	7 (13.2)	0 (0.0)	29 (29.0)
Procedure (Application)	12 (34.3)	37 (69.8)	5 (41.7)	54 (54.0)
Comparison (Products)	0 (0.0)	5 (9.4)	1 (8.3)	6 (6.0)
Before-After Bleaching	5 (14.3)	20 (37.7)	4 (33.3)	29 (29.0)
Post-op sensitivity/Symptom	0 (0.0)	10 (18.9)	1 (8.3)	11 (11.0)
Post-op Experience	4 (11.4)	31 (58.5)	1 (8.3)	36 (36.0)
Commercial	2 (5.7)	3 (5.7)	0 (0.0)	5 (5.0)
Education	0 (0.0)	2 (3.8)	9 (75.0)	11 (11.0)

The information quality for most videos was low, with only 12% classified as ‘good’, 53% as ‘moderate’ and 35% as ‘poor’. There were good inter-rater and intra-rater reliability regarding the evaluation of information quality, respectively, κ = 0.72 κ = 0.80. Videos scored as ‘good’ information quality involved the lower mean number of views (424,774.33), while ‘moderate’ quality videos involved the views with 931,467.28 and ‘poor’ quality videos involved higher mean number of 4,700,479.97 views; however, there were no statistical differences among the groups (*P* > 0.05). Poor-information content videos had a significantly higher number of viewing rates than the other groups (*P* = 0.002), while the duration was significantly higher in moderate content videos (*P* = 0.002). Nevertheless, the number of likes, dislikes, comments did not have significant differences among the groups (*P* > 0.05) (Table 3). There was a moderate correlation between viewers’ interaction index and viewing rate in good information quality group (*r* = 0.648, *P* < 0.05) (Table 4). There was a significant difference between the quality of video information and material type (*P* < 0.001) (Table 5).

**Table 3:** Comparison of video parameters between poor, moderate, and good information content videos about teeth whitening

Parameters	Poor-Information Content (*n* = 35)		Moderate-Information Content (*n* = 53)		Good-Information Content (*n* = 12)		*P*^Ø^
	Mean	SD	Mean	SD	Mean	SD	
Number of Views	4,700,479.97	10,841,998.74	931,467.28	1,454,430.84	424,774.33	512,809.53	.761
Duration (minute)	4.79	3.36	6.80	3.18	4.64	1.93	.002
Number of Likes	26,360.23	62,153.65	12,214.15	21,666.26	3556.83	6554.50	.260
Number of Dislikes	3202.11	7049.03	490.36	731.06	201.17	267.38	.173
Number of Comments	1118.37	3429.55	673.40	968.29	316.33	435.32	.153
Viewers’ Interaction Index	1.05	1.41	1.69	1.68	0.57	0.38	.002
Viewing rate	1,944,029.17	7,476,505.57	285,784.39	1,026,058.46	175,834.36	274,334.33	.002

*SD* Standard deviation, ^Ø^Results of Kruskall-Wallis test

**Table 4:** Spearman correlation coefficients between viewers’ interaction index and viewing rate in different information content groups

		Viewers’ Interaction Index	Viewing Rate
Poor-Information Content	Viewers’ Interaction Index	–	−0.262*
	Viewing Rate	−0.262*	–
Moderate-Information Content	Viewers’ Interaction Index	–	0.054*
	Viewing Rate	0.054*	–
Good-Information Content	Viewers’ Interaction Index	–	0.648**
	Viewing Rate	0.648**	–

Significance levels, *P > 0.05 **P < 0.05

**Table 5:** Comparison of information content of videos and the materials used in the teeth whitening

	Material Type			Total
	Dental	Natural	Other	
Poor Information Content	4	21	10	35
Moderate Information Content	42	2	7	51
Good Information Content	7	0	1	8
Total	53	23	18	94
P*	< 0.001			

*Results of Fischer’s Exact test

## Discussion

The content of YouTube™ videos related to various medical issues such as chronic diseases, epilepsy, hypertension, and multiple sclerosis was evaluated in the literature [3, 23, 24]. Several topics related to oral health and dentistry such as early childhood caries, orthognathic surgery and root canal treatment have been evaluated on YouTube™ [11, 20, 22]. This is the first study to analyze the quality of YouTube™ videos information about teeth whitening.

With the increasing popularity of Internet and social media in recent years, it is possible to assume that YouTube™ is the first advisory platform for patients interested in teeth whitening. YouTube™ provides more engaging visual content than other social media platforms. Accordingly, a significant proportion of patients who need teeth whitening will first look for information on treatment alternatives via YouTube™. The validity of the information on YouTube™ can be questioned as video sharing is simple, and video content is not standardized [25]. Therefore, we aimed to evaluate the quality and content of videos about teeth whitening on YouTube™. We concluded that YouTube™ users showed great interest in videos related to teeth whitening, professionals and non-professionals uploaded a lot of videos, the number of views of these uploaded videos was quite high and viewers often commented on videos to share their experiences and knowledge.

Social media is often a more accessible communication network for patients and makes it easier for patients to obtain the information. Besides, the sharing of subjective thoughts by information sources carries some risks. Incorrect information may be encountered that prevents patients from attempting to access treatment or being referred to alternative treatment sources. YouTube™ does not impose any restriction or content control on medical videos; therefore, the content quality of medical videos is deficient. Patients may also find it challenging to apply information from videos [26]. Clinicians should keep in mind that even if the information given in the videos is correct, patients may not always be able to interpret it correctly.

The viewing rate of videos uploaded to YouTube™ by laypersons was very high. Most of the videos consist of definitions, procedures, and whitening experience. Professional sources of information from specialists, dentists, and dental institutions have been under-represented in our study. Since the referee does not have the information, it may not be evidence-based. Also, most of the YouTube™ content about teeth whitening is irrelevant or missing. Despite these limitations, it has been proven that 33% of people believe that health-related information comes from the most popular sources [25]. The results raise concern, mainly due to the lack of evidence-based information and the contribution of a layperson to YouTube™. This is related to previous studies [22, 27] that have found that most of the videos uploaded on health care issues are from layperson resources.

Good-information content videos are mostly uploaded by professionals; poor and moderate content videos are uploaded by laypersons in the present study. The longest videos were found in the ‘Poor-information content’ category in our study. It was thought that videos that were uploaded by a layperson included different subjects such as their own social life, which prolonged the duration of the videos. Still, the most viewed and liked videos were in the ‘Poor-information content’ resource category. We also found that the viewers’ interaction index was higher in the poor-information content than good-information content. This is because they are easier to understand and more patient-friendly than people with no medical background.

In the present study, most videos had poor and moderate-information quality. This is related to studies evaluating YouTube™ videos on other healthcare issues that are found to be low in content. In our study, most of the top-ranked videos had poor-information content. This means that the relevance applied by YouTube™ does not reflect the actual content of the videos. Most videos have misleading content and limited information, and this increases the risk of spreading incorrect information and may adversely affect patient behavior about teeth whitening.

Teeth whitening is of great interest to people compared to other areas of dentistry. People are so interested in tooth color, and they think their teeth are not white enough [15]. Good-information content videos mostly included dental products, and poor-information content included natural and other products in this study. This is because videos of dental products are uploaded by professionals, natural and other products are uploaded by laypersons. Easy access to natural and other products by people, preparing at home and title with homemade teeth whitening videos attracted the attention of people increased the number of views and likes.

The content of YouTube™ is dynamic; therefore, the search query results vary continuously, because the areas of interest and video viewing times change over time. However, the use of a long working period can produce a large amount of social media data that is often unmanageable and difficult to analyze. On the other hand, it should be kept in mind that YouTube™ variables can be manipulated.

## Conclusion

YouTube™ could not be regarded as a completely reliable source of information for patients about teeth whitening. Professional dental associations, dental clinics, and dentists should upload peer-reviewed videos to YouTube™ about teeth whitening. Further studies are needed to investigate the quality of information about teeth whitening on different social media platforms.

## Data Availability

The datasets used and analyzed during the current study are available from the corresponding author on reasonable request.

## References

[1] AlGhamdi KM, Moussa NA (2012). Internet use by the public to search for health-related information. Int J Med Inform.

[2] Gholami-Kordkheili F, Wild V, Strech D (2013). The impact of social media on medical professionalism: a systematic qualitative review of challenges and opportunities. J Med Internet Res.

[3] Madathil KC, Rivera-Rodriguez AJ, Greenstein JS, Gramopadhye AK (2015). Healthcare information on YouTube: a systematic review. Health Inform J.

[4] Greene JA, Choudhry NK, Kilabuk E, Shrank WH (2011). Online social networking by patients with diabetes: a qualitative evaluation of communication with Facebook. J Gen Intern Med.

[5] McMullan M (2006). Patients using the internet to obtain health information: how this affects the patient-health professional relationship. Patient Educ Couns.

[6] Atkinson NL, Saperstein SL, Pleis J (2009). Using the internet for health-related activities: findings from a national probability sample. J Med Internet Res.

[7] https://www.alexa.com/. Accessed 30 May 2020..

[8] Bezner SK, Hodgman EI, Diesen DL (2014). Pediatric surgery on YouTube™: is the truth out there?. J Pediatr Surg.

[9] Sampson M, Cumber J, Li C, Pound CM, Fuller A, Harrison D (2013). A systematic review of methods for studying consumer health YouTube videos, with implications for systematic reviews. PeerJ..

[10] Butler DP, Perry F, Shah Z, Leon-Villapalos J (2013). The quality of video information on burn first aid available on YouTube. Burns..

[11] Nason K, Donnelly A, Duncan HF (2016). YouTube as a patient-information source for root canal treatment. Int Endod J.

[12] Sorensen JA, Pusz MD, Brietzke SE (2014). YouTube as an information source for pediatric adenotonsillectomy and ear tube surgery. Int J Pediatr Otorhinolaryngol.

[13] Joiner A, Luo W (2017). Tooth colour and whiteness: a review. J Dent.

[14] Herrera A, Martín J, Pérez F (2016). Is personality relevant in the choice of bleaching?. Clin Oral Investig.

[15] Alkhatib MN, Holt R, Bedi R (2004). Prevalence of self-assessed tooth discolouration in the United Kingdom. J Dent.

[16] Shulman JD, Maupome G, Clark DC, Levy SM (2004). Perceptions of desirable tooth color among parents, dentists and children. J Am Dent Assoc..

[17] Montero J, Gómez-Polo C, Santos JA, Portillo M, Lorenzo MC, Albaladejo A (2014). Contributions of dental colour to the physical attractiveness stereotype. J Oral Rehabil.

[18] Tin-Oo MM, Saddki N, Hassan N (2011). Factors influencing patient satisfaction with dental appearance and treatments they desire to improve aesthetics. BMC Oral Health.

[19] Samorodnitzky-Naveh GR, Geiger SB, Levin L (2007). Patients’ satisfaction with dental esthetics. J Am Dent Assoc.

[20] ElKarmi R, Hassona Y, Taimeh D, Scully C (2017). YouTube as a source for parents' education on early childhood caries. Int J Paediatr Dent.

[21] Desai T, Shariff A, Dhingra V, Minhas D, Eure M, Kats M (2013). Is content really king? An objective analysis of the public's response to medical videos on YouTube. PLoS One.

[22] Hegarty E, Campbell C, Grammatopoulos E, DiBiase AT, Sherriff M, Cobourne MT (2017). YouTube™ as an information resource for orthognathic surgery. J Orthod.

[23] Kumar N, Pandey A, Venkatraman A, Garg N (2014). Are video sharing web sites a useful source of information on hypertension?. J Am Soc Hypertens.

[24] Fernandez-Luque L, Elahi N, Grajales FJ (2009). An analysis of personal medical information disclosed in YouTube videos created by patients with multiple sclerosis. Stud Health Technol Inform.

[25] Nason GJ, Tareen F, Quinn F (2013). Hydrocele on the web: an evaluation of internet-based information. Scand J Urol.

[26] Ajumobi AB, Malakouti M, Bullen A, Ahaneku H, Lunsford TN (2016). YouTube™ as a source of instructional videos on bowel preparation: a content analysis. J Cancer Educ.

[27] Özdal Zincir Ö, Bozkurt AP, Gaş S (2019). Potential patient education of YouTube videos related to wisdom tooth surgical removal. J Craniofac Surg.

